# Situating Meditation Apps Within the Ecosystem of Meditation Practice: Population-Based Survey Study

**DOI:** 10.2196/43565

**Published:** 2023-04-28

**Authors:** Sin U Lam, Qiang Xie, Simon B Goldberg

**Affiliations:** 1 Department of Counseling Psychology University of Wisconsin-Madison Madison, WI United States; 2 Center for Healthy Minds University of Wisconsin-Madison Madison, WI United States

**Keywords:** mindfulness, meditation, consumer behavior, user engagement, mobile health, digital health, mobile phone

## Abstract

**Background:**

Meditation apps have the potential to increase access to evidence-based strategies to promote mental health. However, it is currently unclear how meditation apps are situated within the broader landscape of meditation practice and what factors may influence engagement with them.

**Objective:**

This study aimed to clarify the prevalence and correlates of meditation app use in a population-based sample of individuals with lifetime exposure to meditation in the United States. In addition, we sought to identify the concerns and desired features of meditation apps among those with lifetime exposure to meditation.

**Methods:**

A total of 953 participants completed an initial screening survey. Of these 953 participants, 434 (45.5%) reported lifetime exposure to meditation and completed a follow-up survey (434/470, 92.3% response rate) assessing their meditation app use, anxiety, depression, loneliness, initial motivation for meditation, and concerns about and desired features of meditation apps.

**Results:**

Almost half (434/953, 45.5%) of the participants who completed the screening survey reported lifetime exposure to meditation. Among those with lifetime exposure to meditation (ie, meditators), more than half (255/434, 58.8%) had used meditation apps at least once in their lives, and 21.7% (94/434) used meditation apps weekly or daily (ie, active users). Younger age, higher anxiety, and a mental health motivation for practicing meditation were associated with lifetime exposure to meditation apps. Among meditators, those with lifetime exposure to meditation apps were more likely to report concerns about apps, including concerns regarding the cost and effectiveness of apps, time required for use, technical issues with apps, and app user-friendliness. Meditators who used meditation apps weekly or daily (ie, active users) were younger, less likely to be men and non-Latinx White individuals and have lower income, and more likely to have an initial spiritual motivation for meditation. Active users reported more concerns regarding usability and technical problems and were less likely to report disinterest in apps. Headspace and Calm were the most frequently used apps. Tips and reminders for practice, encouragement of “mini” practices, and mental health content were the most desired features. Participants were less interested in social features (eg, the ability to communicate with other users or teachers).

**Conclusions:**

Meditation apps are commonly used by meditators in the United States, with a higher use among certain demographic groups. Future studies may increase user engagement in meditation apps by addressing concerns (eg, cost and effectiveness) and incorporating desired features (eg, tips and reminders for practice).

## Introduction

### Background

There have been dramatic innovations in digital technology over the past 10 years. According to the Pew Research Center, 85% of Americans owned a smartphone as of 2021 compared with only 35% in 2011 [1]. Smartphones are increasingly used to deliver health interventions [2]. Smartphone-delivered interventions may be particularly helpful in expanding access to health services and integrating health support into people’s daily lives [3,4]. The potential for smartphone-delivered interventions has been obvious during the COVID-19 pandemic, with the increasing need for mental health care coupled with a shortage of mental health services [3]. There is growing evidence supporting the efficacy of smartphone-delivered interventions for both physical and mental health [5-10].

*Meditation* is an umbrella term for various forms of mental training focused on the cultivation of attentional and affective regulatory skills that promote well-being [11]. Meditation has become increasingly popular in the United States (US) over the past 10 years. According to the National Health Interview Survey (NHIS) conducted by the Centers for Disease Control and Prevention, the percentage of adults in the US who used meditation in the last 12 months increased from 6.5% in 2012 to 21.1% in 2017 [12,13]. Although there is a wide variety of types of meditation, for the purposes of the NHIS and this study, meditation was operationalized as mindfulness, mantra, and spiritual meditation, which are 3 popular forms [14]. Research suggests that meditation may be beneficial for mental and physical health [15-20].

Advancements in digital technology may have contributed to the increased popularity of meditation over the past 10 years. Meditation practices figure prominently in the mental health app landscape. A recent study found that 2 popular meditation apps (Headspace and Calm) alone accounted for 96% of daily active mental health app users [21]. Moreover, there is meta-analytic evidence suggesting that app-delivered meditation interventions significantly reduce psychological distress and improve quality of life [22].

Although meditation apps potentially increase access to evidence-based psychological strategies and demonstrate promising effects on health outcomes, they are not without limitations. Notably, meditation apps, as with other smartphone-delivered interventions, have notoriously poor user retention, which can limit the interventions’ potential benefits in the long term and may undermine the validity of studies evaluating these interventions [23,24]. Given the potentially negative impact of poor retention, it is important to investigate why people do or do not continue using meditation apps.

Recent studies on meditation apps and other smartphone-delivered interventions have begun exploring perceptions of these interventions, the demographic and clinical characteristics of users, and the role of these factors in dropout and engagement patterns. For example, recent studies suggest that app users frequently report concerns about the security and privacy of apps, which may be barriers to continued use [25,26]. Huberty et al [27] investigated the user characteristics and use patterns of the Calm meditation app among paid subscribers. They found that participating subscribers were mainly White individuals and women, the most common reasons for trying Calm were sleep difficulties and psychological distress, and those with sleep difficulties used Calm more frequently. Although an intriguing first look at associations between user variables and use, the findings of Huberty et al [27] may or may not generalize to those who use other meditation apps given that different meditation apps may have different user bases and features that may or may not affect the findings (eg, the Liberate app, which was specifically designed for Black individuals [28]).

To date, the role of meditation apps within the broader landscape of meditation practice in the US has not been clarified. This is an important area to clarify given the promising effects of meditation training on health [17,29] and the ubiquity of meditation practices within mental health apps [21]. It is currently unclear how frequently meditators are exposed to meditation through apps, who is being exposed in this way, and what factors are associated with persistence with app-based meditation training. In addition, the concerns that meditators may have regarding meditation apps and the features that may be desired by potential users have not been investigated. Insights gained on these topics could collectively be used to inform the ongoing development and dissemination of app-based meditation interventions and maximize the public health impact of these interventions.

### Objectives

To address these questions, we conducted a web-based survey using a population-based sampling method in the US. We had 4 primary aims. First, we sought to provide estimates of the prevalence and patterns of meditation app use in a demographically representative sample of meditators. In this study, *meditators* refers to people with lifetime exposure to NHIS-defined meditation (ie, mindfulness, mantra, or spiritual meditation) [14]. We focused on meditators instead of the general population (ie, meditators and nonmeditators) as we were interested in how meditation apps are situated within the broader landscape of meditation practice and factors that might influence engagement with meditation apps. Second, we aimed to examine the demographic (eg, gender and race or ethnicity) and psychological (eg, anxiety and depression) correlates of meditation app use. Third, we sought to understand which meditation apps are being used by meditators. Finally, we aimed to characterize the concerns and desired features of meditation apps among meditators. Given the limited existing literature, this study was exploratory and we had no a priori hypotheses.

## Methods

### Ethics Approval

The study procedures were approved by the Institutional Review Board of the University of Wisconsin–Madison (reference 2020-1368).

### Participants

We recruited participants using the Prolific platform (Prolific Academic Ltd) [30] in November and December 2020. Prolific is a recruitment platform that has been shown to include participants who are more diverse, less dishonest, and less familiar with research materials than other web-based recruitment platforms (eg, Amazon Mechanical Turk) [31,32]. Using Prolific’s representative sampling procedure, we recruited participants based on their age, sex, and race in proportion to the US Census data.

In this sample, 96% (953/993) of the participants completed our screening survey, passed an attention check (“I have been randomly selecting responses on this survey”), and indicated whether they had practiced meditation in their lifetime. A comparison of the overall sample with the US adult population [33] showed that this sample was more educated (482/953, 50.6% with a bachelor’s degree or higher vs 32.1% in the 2015-2019 US Census), older (median age 44 years vs 38 years), and wealthier (median income US $40,000 vs US $34,103). Although some racial and ethnic groups (Asian, Black, and multiracial) were represented in proportion to the US population, non-Latinx White participants were overrepresented (673/953, 70.6% vs 60.7%), and Latinx participants were underrepresented (57/953, 6% vs 18%). The low Latinx representation was most likely the result of Prolific matching based on race but not ethnicity (for sample demographics, see Multimedia Appendix 1).

Almost half (470/953, 49.3%) of the participants who underwent screening reported exposure to meditation (ie, mindfulness, mantra, or spiritual meditation) at some point in their lifetime. Meditators (ie, those with lifetime exposure to meditation) were invited to complete a follow-up survey that assessed various aspects of their meditation practice, including their experience with meditation apps. Those who completed the follow-up survey and passed a second attention check (“Please select the leftmost response”) formed the primary analytic sample for this study (n=434). This sample represented most of those invited to complete the follow-up survey (434/470, 92.3% response rate). We compared the demographics of those who completed the follow-up survey with the demographics of those who were invited but did not complete the follow-up survey using a correlation coefficient for ease of interpretation. Of note, a correlation between 2 dichotomous variables is a special case of the generalized Pearson correlation coefficient [34]. The results showed that non-Latinx White participants completed the follow-up survey at a higher rate (*r*=0.15; *P*=.001), although no significant differences were found for other demographic variables (age, gender, education, or income; *r*=−0.05 to 0.05; *P>*.28 in all cases).

### Measures

#### Demographics

Participants were asked to provide their age, gender identity, race and ethnicity, highest degree of education, and annual income (for the items assessed, see Multimedia Appendix 2). The following demographic variables were dichotomized as covariates in our models: gender (not men as the reference group), race and ethnicity (racial and ethnic minority as the reference group), education (not college graduate as the reference group), and annual income (income below the US population per capita median [US $34,103] as the reference group [33]).

#### Meditation App Use

Participants who completed the follow-up survey (ie, meditators) were asked about their meditation practice frequency and experience with meditation apps. We assessed the specific meditation apps that meditators used, the specific apps they used the most, and their meditation app use frequency (1=*never*, 2=*several times per year*, 3=*monthly*, 4=*weekly*, and 5=*daily*). Participants were asked to indicate any apps they had used. Options for text entry were provided for participants to indicate meditation apps that were not on the list. The list of meditation apps was adopted from the study by Carlo et al [35] and included 22 popular meditation apps (eg, Headspace, Calm, and Insight Timer; Multimedia Appendix 3).

#### Psychological Measures

On the basis of prior work indicating that individuals with elevated mental health symptoms were more likely to access meditation apps [27] and evidence that elevated symptoms may be associated with a greater risk of adverse responses to meditation, which could decrease persistence [36,37], we assessed the symptoms of depression, anxiety, and loneliness.

The Patient-Reported Outcomes Measurement Information System Depression and Anxiety scales were used to assess depression (eg, “I felt worthless”) and anxiety (eg, “I felt fearful”) symptoms [38]. Participants responded to each item using a 5-point Likert-type scale (1*=never*; 5*=always*). Higher total scores reflected higher levels of anxiety and depressive symptoms. Internal consistency was adequate in this sample (Cronbach α=.93 and .90 for the Patient-Reported Outcomes Measurement Information System Depression and Anxiety scales, respectively).

The 5-item National Institutes of Health Toolbox Loneliness Scale [39] was used to assess loneliness. Participants were asked to rate their experience of loneliness in the past week (eg, “I feel alone”) on a 5-point Likert-type scale (1*=never*; 5*=always*). Internal consistency was adequate (Cronbach α=.94) in this sample.

#### Meditation Motivation

Participants were asked to respond to items adapted from the study by Pepping et al [40] to assess motivation for meditation practice. Participants indicated which factors motivated them to begin their meditation practice. Options included physical health, emotional health or stress reduction, sociocultural and spiritual reasons, and “other” to indicate reasons not listed previously (Multimedia Appendix 3).

#### Concerns About Meditation Apps

To assess participants’ concerns about meditation apps, we adopted 10 items from the studies by Stoyanov et al [41], Torous et al [42,43], and Kenny et al [44]. Example items include “cost of apps,” “I am not interested in them,” “they do not target or help with my goals,” and “technical problems” (Multimedia Appendix 3). Participants could select multiple concerns. We evaluated associations between app use and individual concerns, as well as with the total number of concerns.

#### Desired Features of Meditation Apps

Participants were asked to indicate to what extent they were interested in a list of different meditation app features on a 6-point Likert-type scale (1*=not at all*; 6*=a great deal*). The sample list of suggested items was generated based on relevant criteria from the Mobile App Rating Scale [41] as well as features included in existing apps or that could be included in future apps. Sample items included were “ability to connect with other users,” “having content related to depression and anxiety,” “having tips for daily life practice,” and “the ability to text with a meditation coach.”

### Data Analysis

We first calculated the percentage of meditators (ie, those with lifetime exposure to meditation) who indicated having ever used a meditation app and those reporting active app use (weekly or daily) to provide an estimate of the prevalence of meditation app use among meditators. We then examined sociodemographic (age, gender, race and ethnicity, and income), psychological (depression, anxiety, and loneliness), and motivational (initial motivation for practice) characteristics and concerns regarding meditation apps as correlates of lifetime exposure to meditation apps and active app use. Of note, these analyses were conducted with participants who reported lifetime exposure to meditation and completed the follow-up survey (434/953, 45.5%). Correlations were used to provide a comparable effect size across models given that correlations with dichotomous outcomes or predictors are special cases of the Pearson coefficient [34]. A multiple regression model with non-Latinx as the reference group was conducted to examine associations between active app use and specific racial and ethnic subgroups. In total, 2 sets of sensitivity analyses were conducted: one controlling for demographics and one removing outliers (values 3 SDs from the mean). We used partial correlations for sensitivity analyses by controlling for demographics. Instances where the results changed in the sensitivity analyses were noted. Given that we examined several potential correlates of meditation app use, we controlled for false discovery rate (FDR) using the method by Benjamini and Hochberg [45]. *P* values reported in the text were FDR adjusted [45].

## Results

### Demographics

The meditator sample (ie, those with lifetime exposure to meditation) comprised predominantly women (237/434, 54.6%), with 43.5% (189/434) men and 1.8% (8/434) nonbinary or transgender-identifying participants. Most participants (315/434, 72.6%) identified as non-Latinx White individuals, 12% (52/434) identified as African American individuals, 5.3% (23/434) identified as Latinx individuals, 6.7% (29/434) identified as Asian individuals, 0.5% (2/434) identified as Native American individuals, and 3% (13/434) identified as multiracial. Meditators were, on average, aged 43.77 (SD 15.53) years. The average income was US $54,389.56 (SD US $60,126.77; median US $40,000). Less than half (187/434, 43.1%) of the participants had an annual income below the median for the US population (US $34,103) [33].

Sample descriptive statistics of meditators in this study (434/953, 45.5%) are shown in Multimedia Appendix 1. No continuous variables were found to deviate from normality outside the recommended ranges (ie, skewness <2.00 and kurtosis <7.00 [46]). Specifically, skewness ranged from −1.01 to 0.70 and kurtosis ranged from −1.17 to 0.75 in this study. As reported by Goldberg et al [36], the distribution of lifetime hours of meditation practice was as follows: 0 to 10 (82/434, 18.9%), 11 to 100 (179/434, 41.2%), 101 to 500 (71/434, 16.4%), 501 to 1000 (39/434, 9%), 1001 to 5000 (31/434, 7.1%), and ≥5001 (32/434, 7.4%).

### Meditation App Use

Among meditators (ie, those with lifetime exposure to meditation) who completed the follow-up survey (434/953, 45.5%), more than half (255/434, 58.8%) indicated having used a meditation app in their lifetime. The frequency of current app use among meditators was 7.1% (31/434) daily, 14.5% (63/434) weekly, 10.6% (46/434) monthly, and 20.7% (90/434) several times per year, with the remaining participants reporting either no lifetime exposure to a meditation app (179/434, 41.2%) or no use in the past year (25/434, 5.8%). Mediators with current daily or weekly app use were considered active app users.

Participants with lifetime exposure to meditation apps indicated their most frequently used apps. The most frequently used meditation apps were Headspace (76/255, 29.8%), Calm (73/255, 28.6%), The Mindfulness App (26/255, 10.2%), and Insight Timer (26/255, 10.2%). For those who reported practicing meditation with their most frequently used app weekly, average practice time per week was 48.35 (SD 57.78; range 5 to 420) minutes. For those who reported practicing meditation with their most frequently used app daily, average practice time per day was 24.58 (SD 17.34; range 2 to 90) minutes.

Active app users (weekly or daily use) were predominantly women (61/94, 65%), with 34% (32/94) being men and 1% (1/94) being nonbinary or transgender-identifying individuals. Most active app users (56/94, 60%) identified as non-Latinx White individuals, 22% (21/94) identified as African American individuals, 5% (5/94) identified as Latinx American individuals, 5% (5/94) identified as Asian American individuals, 1% (1/94) identified as Native American individuals, and 6% (6/94) identified as multiracial individuals. The average age of active meditation app users was 40.39 (SD 13.72) years, and the average income was US $67,457 (SD US $84,516; median US $47,500).

### Correlates of Lifetime Exposure to Meditation Apps

**Figure 1:**
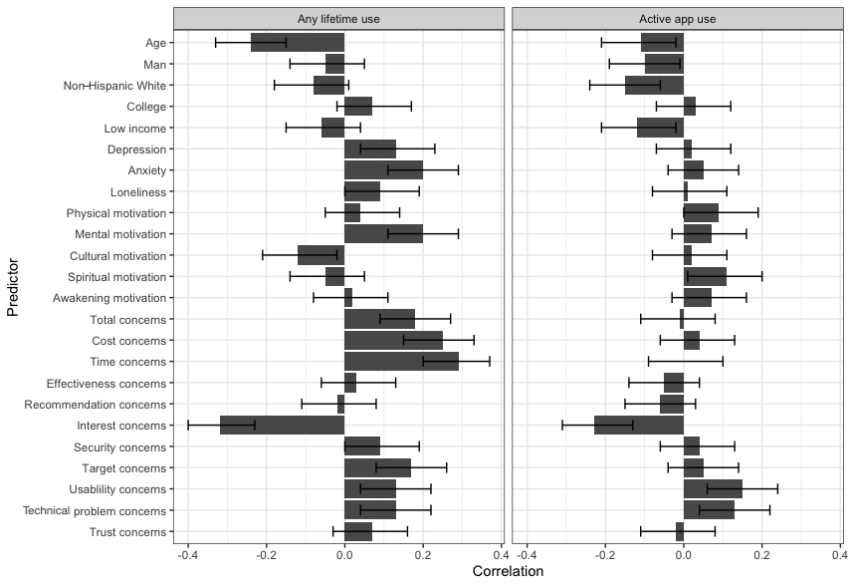
Correlations between participant factors and meditation app use (ie, lifetime and active use; n=434). The bars indicate correlation coefficients with 95% CIs. See Table 1 for these data in tabular format. Active app use: weekly or daily use; awakening motivation: enlightenment, awakening, nirvana, or other ultimate goal motivations for practice; cultural motivation: social, cultural, or religious identity motivations for practice; effectiveness concerns: unsure whether apps are effective; interest concerns: not interested in using apps; mental motivation: mental or emotional health or stress reduction motivations for practice; physical motivation: physical health motivations for practice; recommendation concerns: not recommended by a health care provider; security concerns: concerns regarding the security of health data; target concerns: not targeting or helping with personal goals; technical problem concerns: experiencing technical problems; trust concerns: apps not being a trustworthy source of information; usability concerns: apps not being user-friendly.

Younger participants were more likely to have tried a meditation app (*r*=−0.24; *P*<.001). None of the other sociodemographic variables (ie, gender, race and ethnicity, educational background, and income conditions) was associated with lifetime exposure to meditation apps (Table 1 and Figure 1). Anxiety was correlated with increased likelihood of having used a meditation app (*r*=0.20; *P*<.001). Depression was also correlated with increased likelihood of having used a meditation app (*r*=0.13; *P*=.005), although this association was no longer significant after controlling for demographic variables. Loneliness was not significantly associated with meditation app use (*r*=0.09; *P*=.051). Those who initially practiced meditation for mental health or stress reduction motives were more likely to have used a meditation app (*r*=0.20; *P*<.001). In contrast, individuals who first tried meditation practice for social, cultural, or religious reasons were less likely to try meditation apps (*r*=−0.12; *P*=.02). Other meditation motives were not associated with lifetime exposure to meditation apps: trying meditation for physical health purposes (*r*=0.04; *P*=.36), for general spiritual or self-transformation (*r*=−0.05; *P*=.31), and for achieving other ultimate goals (eg, enlightenment or awakening; *r*=0.02; *P*=.70).

Participants reporting a larger total number of concerns regarding meditation apps were more likely to have used a meditation app (*r*=0.18; *P*=.02). Similarly, most concerns were associated with increased likelihood of having used a meditation app: cost of apps (*r*=0.25; *P*<.001), time required for use (*r*=0.29; *P*<.001), technical issues (*r*=0.17; *P*<.001), not being user-friendly (*r*=0.13; *P*<.001), and not being targeted for personal goals (*r*=0.13; *P*<.001). The only concern that was negatively associated with lifetime exposure to a meditation app was being disinterested in them (*r*=−0.32; *P*<.001). The remaining concerns (doubt about effectiveness, not recommended by a health care provider, trustworthiness of apps as a source of information, and concerns about the security of health data) were not associated with lifetime exposure to meditation apps (*r*=−0.02 to 0.09; *P*>.05 in all cases).

**Table 1:** Correlations between participant factors and meditation app use (n=434).

Variable		Any lifetime use				Active app use		
		*r*	*P* value	FDR^a^-adjusted *P* value	*r*		*P* value	FDR-adjusted *P* value
**Demographics**								
	Age	−0.24	<.001^b^	<.001^b^	−0.11		.02^c^	.03^c^
	Men	−0.05	.32	.27	−0.10		.04^c^	.02^c^
	Non-Latinx White	−0.08	.08	.76	−0.15		.001^d^	.02^c^
	Bachelor’s degree	0.07	.12	.09	0.03		.56	.72
	Low income	−0.06	.25	.16	−0.12		.01^c^	.005^d^
**Psychological factors**								
	Depression	0.13	.005^d^	.07	0.02		.61	.59
	Anxiety	0.20	<.001^b^	.002^d^	0.05		.30	.46
	Loneliness	0.09	.05	.29	0.01		.79	.79
**Initial motivation for meditation**								
	Physical motivation^e^	0.04	.36	.32	0.09		.05	.03^c^
	Mental motivation^f^	0.20	<.001^b^	.003^d^	0.07		.16	.53
	Cultural motivation^g^	−0.12	.02^c^	.047^c^	0.02		.73	.80
	Spiritual motivation^h^	−0.05	.31	.36	0.11		.03^c^	.04^c^
	Awakening motivation^i^	0.02	.70	.48	0.07		.18	.11
**Concerns about meditation apps**								
	Total concerns^j^	0.18	<.001^b^	.001^d^	−0.01		.78	.67
	Cost concerns	0.25	<.001^b^	<.001^b^	0.04		.46	.54
	Time concerns	0.29	<.001^b^	<.001^b^	0.001		.98	.66
	Effectiveness concerns^k^	0.03	.48	.91	−0.05		.29	.26
	Recommendation concerns^l^	−0.02	.72	.83	−0.06		.22	.35
	Interest concerns^m^	−0.32	<.001^b^	<.001^b^	−0.23		<.001^b^	<.001^b^
	Security concerns^n^	0.09	.05	.15	0.04		.47	.85
	Target concerns^o^	0.17	<.001^b^	.002^d^	0.05		.29	.45
	Usability concerns^p^	0.13	.006^d^	.02^c^	0.15		.002^d^	.004^d^
	Technical problem concerns^q^	0.13	.005^d^	.02^c^	0.13		.005^d^	.01^c^
	Trust concerns^r^	0.07	.17	.15	−0.02		.74	.86

^a^FDR: false discovery rate.

^b^*P*<.001.

^c^*P*<.05.

^d^*P*<.01.

^e^Physical motivation: physical health motivations for practice.

^f^Mental motivation: mental or emotional health or stress reduction motivations for practice.

^g^Cultural motivation: social, cultural, or religious identity motivations for practice.

^h^Spiritual motivation: general spiritual or self-transformation motivations for practice.

^i^Awakening motivation: enlightenment, awakening, nirvana, or other ultimate goal motivations for practice.

^j^Total concerns: the total number of concerns.

^k^Effectiveness concerns: unsure whether apps are effective.

^l^Recommendation concerns: not recommended by a health care provider.

^m^Interest concerns: not interested in using apps.

^n^Security concerns: concerns regarding the security of health data.

^o^Target concerns: not targeting or helping with personal goals.

^p^Usability concerns: apps not being user-friendly.

^q^Technical problem concerns: experiencing technical problems.

^r^Trust concerns: apps not being a trustworthy source of information.

### Correlates of Active App Use

Correlational analysis results surviving FDR *P* value correction indicated that active app users (weekly or daily) were less likely to be non-Latinx White (*r*=−0.15; *P*<.001). Active app users were also less likely to indicate that they were disinterested in meditation apps (*r*=−0.23; *P*<.001) and more likely to endorse concerns regarding apps not being user-friendly (*r*=0.15; *P*=.002) and having technical problems (*r*=0.13; *P*=.005). Other demographic factors such as age, gender, education, and income were not associated with active app use. The associations between active app use and other concerns regarding meditation apps, the total number of concerns, and motives for meditation practices did not survive *P* value correction (Table 1).

To further examine the racial and ethnic demographics of active meditation app users, a multiple regression model was conducted using non-Latinx White participants as a reference group. The results showed that Black (β=.18; *P*<.001) and multiracial (β=.12; *P*=.01) participants were more likely to be active app users relative to non-Latinx White participants. This effect persisted after controlling for age, gender, education, and income status (β=.16 and .11 and *P*=.001 and .02 for Black and multiracial participants relative to non-Latinx White participants, respectively). There were no significant differences between other racial and ethnic groups and non-Latinx White participants in terms of active use of meditation apps (Table 2).

**Table 2:** Associations between demographic variables and active app use (n=434).

Demographics			β (SE^a^)		95% CI		*P* value
**Race and ethnicity^b^**							
	Black	.16 (0.05)		0.07 to 0.25		.001	
	Latinx	.01 (0.05)		−0.09 to 0.10		.90	
	Asian	−.04 (0.05)		−0.13 to 0.06		.46	
	Native American	.03 (0.05)		−0.06 to 0.13		.47	
	Multiracial	.11 (0.05)		0.02 to 0.21		.02	
Age			−.11 (0.05)		−0.21 to −0.01		.03
Men			−.11 (0.05)		−0.20 to −0.02		.02
Low income			−.16 (0.05)		−0.26 to −0.06		.002
College			.00 (0.05)		−0.10 to −0.10		.95

^a^SE rounded to 2 digits.

^b^Non-Latinx White participants as a reference group.

### Concerns About Meditation Apps

**Figure 2:**
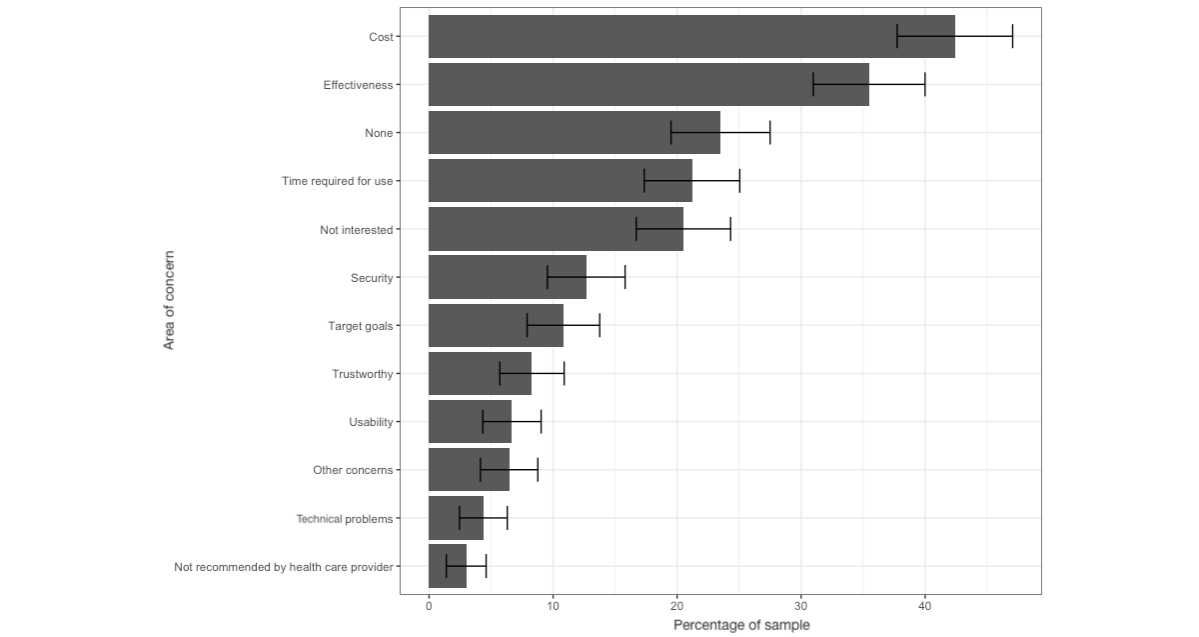
Concerns about meditation apps among meditators (n=434). The bars indicate the percentage of the sample reporting a given concern along with 95% CIs. Effectiveness: unsure whether apps are effective; security: concerns regarding the security of health data; target goals: not targeting or helping with personal goals; technical problems: experiencing technical problems; trustworthy: apps not being a trustworthy source of information; usability: apps not being user-friendly.

The most common concern regarding meditation apps among meditators was the cost of the apps (184/434, 42.4%; Figure 2). Doubts regarding app effectiveness (154/434, 35.5%), time required for use (92/434, 21.2%), and lack of interest (89/434, 20.5%) were also commonly endorsed. Fewer participants endorsed concerns about app usability (ie, not being user-friendly; 29/434, 6.7%), technical problems (19/434, 4.4%), and apps not being recommended by a health care provider (13/434, 3%; Figure 2; Multimedia Appendix 4).

### Desired Features of Meditation Apps

**Figure 3:**
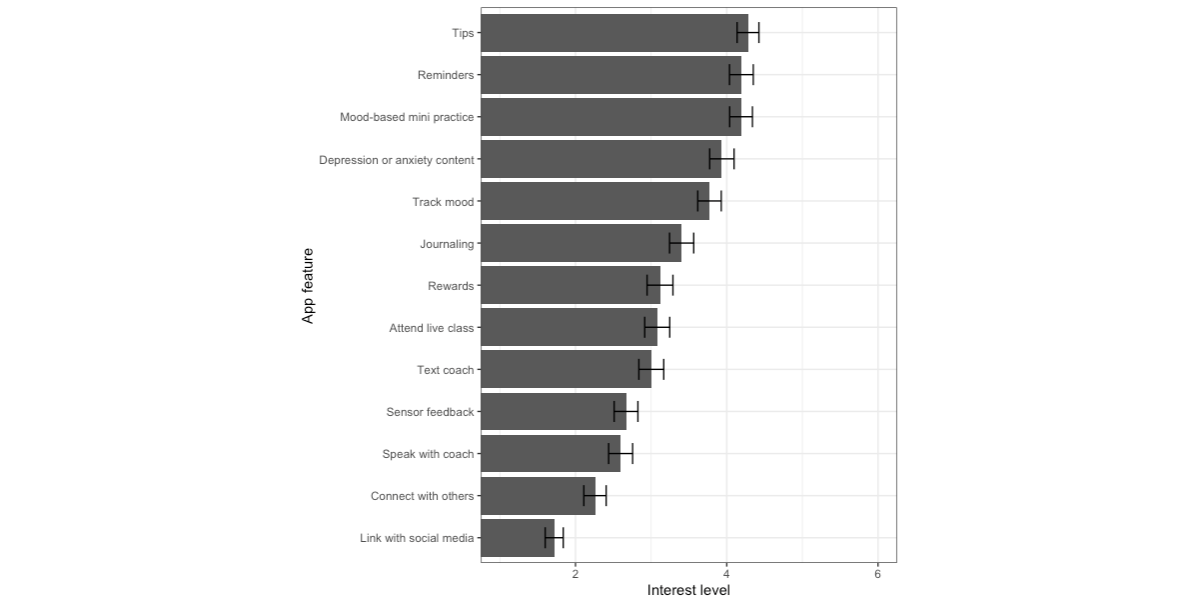
Desired features of meditation apps among meditators (n=434). Interest level was rated from 1 (not at all) to 6 (a great deal). The bars indicate the mean ratings with 95% CIs. Connect with others: ability to connect with other users; depression or anxiety content: content related to depression and anxiety; journaling: opportunity to journal or complete reflections about the experience; link with social media: ability to link app use to social media; mood-based mini practice: encouragement to try mini meditation practices based on mood; reminders: ability to set practice reminders; rewards: rewards (eg, trophies) for practicing for a certain number of days; sensor feedback: automated feedback based on sensors in phone (eg, location, SMS text messages, and camera); speak with coach: ability to speak with a meditation coach; text coach: ability to text with a meditation coach; tips: tips for daily life practice; track mood: complete questionnaires that track mood to customize practices.

Meditators reported the extent to which they desired various features (1*=not at all*; 6*=a great deal*) within meditation apps (Figure 3; Multimedia Appendix 4). Among the list of desired features, the most highly rated included having tips for daily life practice (mean 4.28, SD 1.53), the ability to set reminders to practice (mean 4.19, SD 1.67), encouragement to try “mini” meditation practices based on one’s mood (mean 4.19, SD 1.60), providing content related to depression and anxiety (mean 3.93, SD 1.71), and functions to track mood for customized practices (mean 3.77, SD 1.63). Less highly rated features included automated feedback based on phone sensors (mean 2.67, SD 1.67) and the ability to speak with a meditation coach by phone or video (mean 2.60, SD 1.68). The lowest-rated app features were the ability to connect with other users (mean 2.26, SD 1.57) and the ability to link use to social media (eg, Facebook, Twitter, Instagram, and TikTok; mean 1.72, SD 1.27).

## Discussion

### Principal Findings

This study sought to characterize the current status of meditation apps within the broader context of meditation practice using a population-based sampling method. We examined the prevalence of meditation app use, correlates of use, and concerns and desired features of meditation apps among meditators (ie, those with lifetime exposure to meditation). To contextualize our sample, it is worth comparing sample demographics with those of meditators from nationally representative surveys. Data on the demographics of those with past-year exposure to meditation were reported for the 2012 NHIS [47]. Of note, this is not a perfect comparison given the differing definitions of meditation practice (ie, past-year exposure in the NHIS vs lifetime exposure in this sample) as well as the timing of data collection (2012 vs 2020). However, both samples were of similar age (average age of 43.77, SD 15.53 years in this sample vs most participants being aged between 25 and 64 years in the 2012 NHIS), were mostly college educated (247/434, 56.9% in this sample vs 61.19% in the 2012 NHIS), comprised mostly women (237/434, 54.6% in this sample vs 51.35% in the 2012 NHIS), and had a similar proportion of Black individuals (52/434, 11.98% in this sample vs 12.37% in the 2012 NHIS). In contrast, this sample had fewer Latinx individuals (23/434, 5.3% vs 15.40% in the 2012 NHIS), which may have been due to Prolific matching on race but not ethnicity, as noted previously. The largest difference between the samples was in the proportion of individuals who reported practicing meditation, with 49.3% (470/953) of this screening survey sample reporting lifetime exposure to NHIS-defined meditation categories versus 4.1% with past-year exposure in the 2012 NHIS. This difference may be due to Prolific participants differing from the general population in ways not captured by Prolific’s representative sampling feature. It may also be attributed to the difference in the time frame of the measures (ie, lifetime vs past-year exposure) or reflect the increase in meditation use over the past decade [48].

A striking finding that emerged was the frequency of app use among meditators. Most meditators (255/434, 58.8%) had used a meditation app at least once in their lives, and approximately 1 in 5 (94/434, 21.7%) used meditation apps weekly or more frequently. Thus, it appears that meditation apps are a major component of meditation practice for meditators in the US. The wide use of meditation apps among meditators in the US mirrors the dramatic rise in the adoption of mobile technology generally (eg, smartphones and social media) [49,50] as well as the prominence of meditation content within mental health apps [21]. We also found that active app users were predominantly women (61/94, 65%) and non-Latinx White (56/94, 60%) individuals. This is consistent with the study by Huberty et al [27], who reported that participating subscribers of the Calm app were also predominantly women (79.94%) and White (81.41%) individuals.

Several participant characteristics were associated with increased likelihood of lifetime exposure to meditation apps and active app use. Younger age, higher anxiety, and an initial mental health motivation were associated with greater likelihood of lifetime meditation app use. It may not be surprising that younger people are more likely to try meditation apps given that younger people are more likely to own smartphones and use the internet [51]. The finding that those with higher anxiety and mental health motivations are more likely to try meditation apps is consistent with prior work indicating that mental health concerns are associated with meditation use generally [52].

An intriguing finding was a pattern of increased concerns regarding meditation apps among lifetime meditation app users. We saw this pattern both for the total number of concerns and for endorsement of several specific concerns, including cost, time required, technical issues, user-friendliness, and not being targeted for personal goals. This finding was unexpected given the assumption that these concerns would discourage participants from using meditation apps. On the basis of the observed pattern of findings, these concerns are presumably the result rather than the cause of meditation app use, with individuals who have used these tools finding them lacking in these specific ways. This may be a reasonable possibility given that individuals who have used meditation apps may have a more realistic (and concerning) sense of the cost, time required, and user-friendliness of these apps. In contrast, the only concern that was negatively associated with lifetime exposure to meditation apps was a lack of interest in using them.

Patterns differed somewhat when examining correlates of active app use (weekly or daily). Active users were also younger but less likely to be men, have a low income, and be non-Latinx White individuals. The association with race and ethnicity is particularly intriguing given evidence that racial and ethnic minority populations face more barriers to receiving mental health services [53-55] and tend to access mental health care less frequently than non-Latinx White individuals [56]. Higher active use of meditation apps among racial and ethnic minority participants supports the notion that meditation apps, along with other technology-based interventions, may reduce barriers to use (eg, transportation, time, cost, and stigma) [57]. Unlike associations with lifetime app use, the total number of concerns and concerns regarding cost, time, and the apps being targeted to users’ concerns were not associated with the likelihood of active app use. Having an interest in apps and concerns regarding usability and technical problems remained associated with active app use. Thus, it appears that cost and time concerns may not be barriers for meditators to use meditation apps actively (ie, on a weekly or daily basis).

Mediators expressed various concerns, with concerns regarding app cost and effectiveness being the most common. The concerns about cost mirror the findings of a previous national survey indicating that cost is one of the most important reasons for not downloading health apps [58]. The fact that many meditators (154/434, 35.5%) had concerns about the effectiveness of apps is also notable, particularly given the growing body of meta-analytic evidence suggesting that meditation apps are indeed effective in reducing common mental health symptoms and improving well-being [22]. The fact that many meditators (and perhaps the public generally) are not aware of this research evidence highlights the need for public health education efforts. Such efforts could take cues from health care organizations such as the Veterans Affairs system that, for over a decade, have highlighted the evidence-based nature of the interventions they promote [59].

In terms of desired features, meditators were interested in lighter-weight features, such as tips and reminders for practice and encouragement to engage in brief “mini” practices. This finding aligns with previous research documenting participants’ interest in reminders in health apps [60]. Meditators were also interested in mental health content (depression, anxiety, and mood tracking), which aligned with links between both mental health concerns and motivations and lifetime exposure to meditation apps. In contrast, interest in social components was low. Specifically, meditators were less interested in linking use to social media and connecting with other users and meditation teachers through texting or calling. This finding contradicts prior work demonstrating that social factors such as subjective norms (ie, perception of others’ attitudes on meditation practice) predict meditation app use [61]. A potential explanation for this discrepancy may be the difference in relationship closeness. Specifically, participants in this study reported low interest in connecting with individuals with whom they were likely less close (ie, meditation teachers and other app and social media users), whereas participants in the study by Crandall et al [61] reported their significant others’ perceptions of meditation practice. It is possible that participants perceive meditation as a private or even solitary health behavior, which limits their interest in linking use to social media and connecting with other users and meditation teachers. At the same time, it is important to acknowledge that even features rated as less desirable in this study may in fact be welcome or helpful to certain meditation app users. For instance, several popular meditation apps include these low-rated components (eg, ability to connect with others in Insight Timer and ability to connect with meditation teachers in Ten Percent Happier) [62,63].

### Limitations

Several limitations of this study are noteworthy. First, we conducted a web-based survey, which restricted participants to those with access to the internet. Moreover, our study focused on individuals with lifetime exposure to meditation as defined by the NHIS (ie, mindfulness, mantra, or spiritual meditation), and our sample demographics did not align perfectly with the general US demographics. Therefore, findings may not be generalizable to meditation-naive individuals, those who practice other types of meditation, and the general US population. In addition, the data for app use were collected based on self-report measures, which were susceptible to retrospective and social desirability biases [64]. The results may have been different if app use data had been tracked objectively [65]. Another important limitation was that we assessed a limited number of potential concerns and desired features. It is possible that some important concerns and desired features were not adequately captured. Similarly, we examined only a small set of potential predictors (eg, demographics, anxiety, depression, loneliness, motivation, and concerns about apps) of meditation app use. There are surely many important participant and app-specific characteristics that were not evaluated.

### Future Directions

Given the widespread use of meditation apps among meditators, an important future direction is the additional assessment of meditation app use in national surveys that investigate complementary and integrative health use (eg, NHIS) [66]. Such data could provide a more trustworthy depiction of the role of these technologies than the one this study could provide. A second future direction would involve systematically examining whether the desired features (eg, tips and reminders for practice) are included in publicly available apps (eg, Headspace and Calm). Such a study could empirically assess which features are associated with user ratings of and persistence in using these apps. For example, Huberty et al [27] evaluated the mood check-in feature of the Calm app and found that the use of this feature was associated with higher meditation practice frequency in Calm users, particularly for inactive users. Further observational and experimental studies can be conducted to identify baseline characteristics related to persistence in using meditation apps, as has been done previously [61]. Given that cost is a potentially major barrier to the use of meditation apps, efforts should be made to develop viable cost models for meditation apps. Potential ways of addressing this issue include reimbursement through insurance and increasing awareness of free apps [67]. The intriguing finding that racial and ethnic minority meditators may be more likely to use apps more actively than non-Latinx White participants supports ongoing efforts to develop culturally congruent digital tools [68]. Regarding concerns about the effectiveness of meditation apps, it will be essential to continue conducting rigorous randomized controlled trials to evaluate app effectiveness, with the results of these trials communicated to potential users through public health campaigns. In addition, consumer confidence in digital health technologies may increase when these technologies receive approval from the Food and Drug Administration [69].

## Appendix

Multimedia Appendix 1 Full and follow-up survey sample demographics.

Multimedia Appendix 2 Screening survey items.

Multimedia Appendix 3 Follow-up survey items.

Multimedia Appendix 4 Descriptive statistics for meditators (ie, lifetime exposure to meditation) who completed the follow-up survey (n=434).

## Abbreviations

FDR: false discovery rate
NHIS: National Health Interview Survey

## References

[1] (2021). Mobile fact sheet. Pew Research Center.

[2] Powell AC, Landman AB, Bates DW (2014). In search of a few good apps. JAMA.

[3] Figueroa CA, Aguilera A (2020). The need for a mental health technology revolution in the COVID-19 pandemic. Front Psychiatry.

[4] Nahum-Shani I, Smith SN, Spring BJ, Collins LM, Witkiewitz K, Tewari A, Murphy SA (2018). Just-in-time adaptive interventions (JITAIs) in mobile health: key components and design principles for ongoing health behavior support. Ann Behav Med.

[5] Dounavi K, Tsoumani O (2019). Mobile health applications in weight management: a systematic literature review. Am J Prev Med.

[6] Gandhi S, Chen S, Hong L, Sun K, Gong E, Li C, Yan LL, Schwalm J (2017). Effect of mobile health interventions on the secondary prevention of cardiovascular disease: systematic review and meta-analysis. Can J Cardiol.

[7] Goldberg SB, Lam SU, Simonsson O, Torous J, Sun S (2022). Mobile phone-based interventions for mental health: a systematic meta-review of 14 meta-analyses of randomized controlled trials. PLOS Digit Health.

[8] Lecomte T, Potvin S, Corbière M, Guay S, Samson C, Cloutier B, Francoeur A, Pennou A, Khazaal Y (2020). Mobile apps for mental health issues: meta-review of meta-analyses. JMIR Mhealth Uhealth.

[9] McCarroll R, Eyles H, Ni Mhurchu C (2017). Effectiveness of mobile health (mHealth) interventions for promoting healthy eating in adults: a systematic review. Prev Med.

[10] Xie Q, Torous J, Goldberg SB (2022). E-mental health for people with personality disorders: a systematic review. Curr Psychiatry Rep.

[11] Lutz A, Slagter HA, Dunne JD, Davidson RJ (2008). Attention regulation and monitoring in meditation. Trends Cogn Sci.

[12] (2013). 2012 National Health Interview Survey (NHIS) adult complementary and alternative medicine public use file (ALT). National Center for Health Statistics.

[13] (2018). 2017 National Health Interview Survey (NHIS) Sample Adult Public Use File (samadult). National Center for Health Statistics.

[14] Stussman BJ, Bethell CD, Gray C, Nahin RL (2013). Development of the adult and child complementary medicine questionnaires fielded on the National Health Interview Survey. BMC Complement Altern Med.

[15] Creswell JD, Lindsay EK, Villalba DK, Chin B (2019). Mindfulness training and physical health: mechanisms and outcomes. Psychosom Med.

[16] Dunn TJ, Dimolareva M (2022). The effect of mindfulness-based interventions on immunity-related biomarkers: a comprehensive meta-analysis of randomised controlled trials. Clin Psychol Rev.

[17] Goldberg SB, Riordan KM, Sun S, Davidson RJ (2022). The empirical status of mindfulness-based interventions: a systematic review of 44 meta-analyses of randomized controlled trials. Perspect Psychol Sci.

[18] Gonçalves JPB, Lucchetti G, Menezes PR, Vallada H (2015). Religious and spiritual interventions in mental health care: a systematic review and meta-analysis of randomized controlled clinical trials. Psychol Med.

[19] Lynch J, Prihodova L, Dunne PJ, Carroll Á, Walsh C, McMahon G, White B (2018). Mantra meditation for mental health in the general population: a systematic review. Eur J Integrative Med.

[20] Xie Q, Guan Y, Hofmann SG, Jiang T, Liu X (2023). The potential mediating role of anxiety sensitivity in the impact of mindfulness training on anxiety and depression severity and impairment: a randomized controlled trial. Scand J Psychol.

[21] Wasil AR, Gillespie S, Patel R, Petre A, Venturo-Conerly KE, Shingleton RM, Weisz JR, DeRubeis RJ (2020). Reassessing evidence-based content in popular smartphone apps for depression and anxiety: developing and applying user-adjusted analyses. J Consult Clin Psychol.

[22] Gál É, Ștefan S, Cristea IA (2021). The efficacy of mindfulness meditation apps in enhancing users' well-being and mental health related outcomes: a meta-analysis of randomized controlled trials. J Affect Disord.

[23] Baumel A, Muench F, Edan S, Kane JM (2019). Objective user engagement with mental health apps: systematic search and panel-based usage analysis. J Med Internet Res.

[24] Torous J, Lipschitz J, Ng M, Firth J (2020). Dropout rates in clinical trials of smartphone apps for depressive symptoms: a systematic review and meta-analysis. J Affect Disord.

[25] Torous J, Wisniewski H, Liu G, Keshavan M (2018). Mental health mobile phone app usage, concerns, and benefits among psychiatric outpatients: comparative survey study. JMIR Ment Health.

[26] Zhou L, Bao J, Watzlaf V, Parmanto B (2019). Barriers to and facilitators of the use of mobile health apps from a security perspective: mixed-methods study. JMIR Mhealth Uhealth.

[27] Huberty J, Vranceanu A, Carney C, Breus M, Gordon M, Puzia ME (2019). Characteristics and usage patterns among 12,151 paid subscribers of the calm meditation app: cross-sectional survey. JMIR Mhealth Uhealth.

[28] Owings-Fonner N (2022). Wellness apps designed for people of color. American Psychological Association.

[29] Galante J, Galante I, Bekkers M, Gallacher J (2014). Effect of kindness-based meditation on health and well-being: a systematic review and meta-analysis. J Consult Clin Psychol.

[30] Stanton K, Carpenter RW, Nance M, Sturgeon T, Villalongo Andino M (2022). A multisample demonstration of using the prolific platform for repeated assessment and psychometric substance use research. Exp Clin Psychopharmacol.

[31] Palan S, Schitter C (2018). Prolific.ac—a subject pool for online experiments. J Behavioral Experimental Finance.

[32] Peer E, Brandimarte L, Samat S, Acquisti A (2017). Beyond the Turk: alternative platforms for crowdsourcing behavioral research. J Experimental Soc Psychol.

[33] Quick facts. United States Census Bureau.

[34] Cohen J, Cohen P, West S, Aiken L (2013). Applied Multiple Regression/Correlation Analysis for the Behavioral Sciences (3rd edition).

[35] Carlo AD, Hosseini Ghomi R, Renn BN, Areán PA (2019). By the numbers: ratings and utilization of behavioral health mobile applications. NPJ Digit Med.

[36] Goldberg SB, Lam SU, Britton WB, Davidson RJ (2022). Prevalence of meditation-related adverse effects in a population-based sample in the United States. Psychother Res.

[37] Schlosser M, Sparby T, Vörös S, Jones R, Marchant NL (2019). Unpleasant meditation-related experiences in regular meditators: prevalence, predictors, and conceptual considerations. PLoS One.

[38] Pilkonis PA, Choi SW, Reise SP, Stover AM, Riley WT, Cella D, PROMIS Cooperative Group (2011). Item banks for measuring emotional distress from the patient-reported outcomes measurement information system (PROMIS®): depression, anxiety, and anger. Assessment.

[39] Cyranowski JM, Zill N, Bode R, Butt Z, Kelly MA, Pilkonis PA, Salsman JM, Cella D (2013). Assessing social support, companionship, and distress: National Institute of Health (NIH) Toolbox Adult Social Relationship Scales. Health Psychol.

[40] Pepping CA, Walters B, Davis PJ, O’Donovan A (2016). Why do people practice mindfulness? An investigation into reasons for practicing mindfulness meditation. Mindfulness.

[41] Stoyanov SR, Hides L, Kavanagh DJ, Zelenko O, Tjondronegoro D, Mani M (2015). Mobile app rating scale: a new tool for assessing the quality of health mobile apps. JMIR Mhealth Uhealth.

[42] Torous J, Staples P, Shanahan M, Lin C, Peck P, Keshavan M, Onnela J (2015). Utilizing a personal smartphone custom app to assess the patient health questionnaire-9 (PHQ-9) depressive symptoms in patients with major depressive disorder. JMIR Ment Health.

[43] Torous J, Kiang MV, Lorme J, Onnela J (2016). New tools for new research in psychiatry: a scalable and customizable platform to empower data driven smartphone research. JMIR Ment Health.

[44] Kenny R, Dooley B, Fitzgerald A (2015). Feasibility of "CopeSmart": a telemental health app for adolescents. JMIR Ment Health.

[45] Thissen D, Steinberg L, Kuang D (2016). Quick and easy implementation of the Benjamini-Hochberg procedure for controlling the false positive rate in multiple comparisons. J Educational Behavioral Stat.

[46] Curran PJ, West SG, Finch JF (1996). The robustness of test statistics to nonnormality and specification error in confirmatory factor analysis. Psychological Method.

[47] Burke A, Lam CN, Stussman B, Yang H (2017). Prevalence and patterns of use of mantra, mindfulness and spiritual meditation among adults in the United States. BMC Complement Altern Med.

[48] Lam SU, Riordan KM, Simonsson O, Davidson RJ, Goldberg SB (2023). Who sticks with meditation? Rates and predictors of persistence in a population-based sample in the USA. Mindfulness.

[49] Perrin A (2015). Social media usage: 2005-2015. Pew Research Center.

[50] Poushter J (2016). Smartphone ownership and internet usage continues to climb in emerging economies. Pew Research Center.

[51] Silver L (2019). 2. In emerging economies, smartphone adoption has grown more quickly among younger generations. Pew Research Center.

[52] Cramer H, Hall H, Leach M, Frawley J, Zhang Y, Leung B, Adams J, Lauche R (2016). Prevalence, patterns, and predictors of meditation use among US adults: a nationally representative survey. Sci Rep.

[53] Hines-Martin V, Malone M, Kim S, Brown-Piper A (2003). Barriers to mental health care access in an African American population. Issues Ment Health Nurs.

[54] Miranda R, Soffer A, Polanco-Roman L, Wheeler A, Moore A (2015). Mental health treatment barriers among racial/ethnic minority versus white young adults 6 months after intake at a college counseling center. J Am Coll Health.

[55] Sorkin DH, Murphy M, Nguyen H, Biegler KA (2016). Barriers to mental health care for an ethnically and racially diverse sample of older adults. J Am Geriatr Soc.

[56] Cook BL, Trinh N, Li Z, Hou SS, Progovac AM (2017). Trends in racial-ethnic disparities in access to mental health care, 2004-2012. Psychiatr Serv.

[57] Price M, Yuen EK, Goetter EM, Herbert JD, Forman EM, Acierno R, Ruggiero KJ (2014). mHealth: a mechanism to deliver more accessible, more effective mental health care. Clin Psychol Psychother.

[58] Krebs P, Duncan DT (2015). Health app use among US mobile phone owners: a national survey. JMIR Mhealth Uhealth.

[59] Karlin BE, Cross G (2014). From the laboratory to the therapy room: national dissemination and implementation of evidence-based psychotherapies in the U.S. Department of Veterans Affairs Health Care System. Am Psychol.

[60] Thornton LK, Kay-Lambkin FJ (2018). Specific features of current and emerging mobile health apps: user views among people with and without mental health problems. Mhealth.

[61] Crandall A, Cheung A, Young A, Hooper AP (2019). Theory-based predictors of mindfulness meditation mobile app usage: a survey and cohort study. JMIR Mhealth Uhealth.

[62] Best NI, Durham CF, Woods-Giscombe C, Waldrop J (2020). Combating compassion fatigue with mindfulness practice in military nurse practitioners. J Nurse Practitioner.

[63] Truhlar L, Durand C, Cooper M, Goldsmith C (2022). Exploring the effects of a smartphone-based meditation app on stress, mindfulness, well-being, and resilience in pharmacy students. Am J Health Syst Pharm.

[64] Schwarz N (1999). Self-reports: how the questions shape the answers. Am Psychologist.

[65] Parry DA, Davidson BI, Sewall CJ, Fisher JT, Mieczkowski H, Quintana DS (2021). A systematic review and meta-analysis of discrepancies between logged and self-reported digital media use. Nat Hum Behav.

[66] Clarke TC, Barnes PM, Black LI, Stussman BJ, Nahin RL (2018). Use of yoga, meditation, and chiropractors among U.S. adults aged 18 and over. NCHS Data Brief.

[67] Raber I, McCarthy CP, Yeh RW (2019). Health insurance and mobile health devices: opportunities and concerns. JAMA.

[68] Spanhel K, Balci S, Feldhahn F, Bengel J, Baumeister H, Sander LB (2021). Cultural adaptation of internet- and mobile-based interventions for mental disorders: a systematic review. NPJ Digit Med.

[69] Shuren J, Patel B, Gottlieb S (2018). FDA regulation of mobile medical apps. JAMA.

