# Supplementary material for: Situating Meditation Apps Within the Ecosystem of Meditation Practice: Population-Based Survey Study
Source: JMIR Ment Health. 2023 Apr 28;10:e43565. doi: 10.2196/43565 (PMC10182467; doi:10.2196/43565)
Supplement: Multimedia Appendix 1 [file mental_v10i1e43565_app1.docx]

*Full and Follow-Up Survey Sample Demographics*

|  |  | Full Sample (*n* = 953) | Follow-Up Survey Sample (*n* = 434) |
| --- | --- | --- | --- |
| Variables |  | Mean / Median / % (SD / n) | Mean / Median / % (SD / n) |
| Age | Mean (SD) | 44.69 (16.08) | 43.77 (15.53) |
|  | Median | 44.00 | 42.00 |
| Income | Mean (SD) | $53,762.05 (52150.67) | $54,389.56 (60126.77) |
|  | Median | $40,000 | $40,000 |
|  | Low % (n) | 41.66 (397) | 43.09 (187) |
|  | High % (n) | 58.34 (556) | 56.91 (247) |
|  | $50k or less % (n) | 59.92 (571) | 61.06 (265) |
|  | $50-100k % (n) | 28.54 (272) | 29.03 (126) |
|  | $100-150k % (n) | 7.24 (69) | 4.84 (21) |
|  | $150k+ % (n) | 4.30 (41) | 5.07 (22) |
| Race/Ethnicity | White % (n) | 70.62 (673) | 72.58 (315) |
|  | Black % (n) | 12.80 (122) | 11.98 (52) |
|  | Latinx % (n) | 5.98 (57) | 5.30 (23) |
|  | Asian % (n) | 6.93 (66) | 6.68 (29) |
|  | Native % (n) | 0.42 (4) | 0.46 (2) |
|  | Multiracial % (n) | 3.25 (31) | 3.00 (13) |
| Gender | Female % (n) | 50.47 (481) | 54.61 (237) |
|  | Male % (n) | 48.27 (460) | 43.55 (189) |
|  | Non-binary gender % (n) | 1.26 (12) | 1.84 (8) |
| Transgender | No % (n) | 99.37 (947) | 98.62 (428) |
|  | Yes % (n) | 0.63 (6) | 1.38 (6) |
| College | No % (n) | 49.42 (471) | 43.09 (187) |
|  | Yes % (n) | 50.58 (482) | 56.91 (247) |

*Note*. Low / high income = below or above the US median household income; White = non-Latinx White; College = bachelor’s degree or higher.
