# Supplementary material for: Situating Meditation Apps Within the Ecosystem of Meditation Practice: Population-Based Survey Study
Source: JMIR Ment Health. 2023 Apr 28;10:e43565. doi: 10.2196/43565 (PMC10182467; doi:10.2196/43565)
Supplement: Multimedia Appendix 2 [file mental_v10i1e43565_app2.docx]

*Screening Survey Items*

1. What is your gender?
2. Male
3. Female
4. Transgender (male to female)
5. Transgender (female to male)
6. Non-binary gender
7. Other (please describe)
8. What is your age? ___ years (numeric entry)
9. What is your race? (check all that apply)
10. African American
11. Asian American
12. White
13. Native American / Pacific Islander / Alaskan Native / First Nations
14. Non-US Asian
15. Non-US African
16. Other: _________
17. What is your ethnicity? (check all that apply)
18. Hispanic or Latino
19. Not Hispanic or Latino
20. What is the highest level of education you have completed? (select one)
21. Some high school or less education
22. High school graduate or equivalent (e.g., GED)
23. Some college / community college degree (Associates)
24. Four-year college graduate
25. Masters degree
26. Doctorate (PhD, EdD, PsyD)
27. Medical degree (MD)
28. Other (please describe)
29. What is your best estimate of your total income from all sources, before taxes, in the last year in US dollars? (numeric entry)
30. Have you ever tried any of the following types of meditation, even just once? (select all that apply)
    1. Mantra meditation, including Transcendental Meditation®, Relaxation Response, or Clinically Standardized Meditation?
    2. Mindfulness meditation, including Vipassana, Zen Buddhist meditation, Mindfulness-Based Stress Reduction, and Mindfulness-Based Cognitive Therapy
    3. Spiritual meditation including centering prayer and contemplative meditation
    4. Tibetan meditation including compassion, visualization, analytical meditation, Dzogchen, and Mahamudra
    5. Yogic meditation, including kundalini, pranayama, and chakra meditation
    6. Meditation as part of yoga, qi gong, or tai-chi
    7. Other meditation practices including lovingkindness and body scan
    8. Other type of meditation (please specify) (text field)
    9. I have not tried any type of meditation
31. I have been randomly selecting responses on this survey. (1 = strongly disagree, 5 = strongly agree)
