# Supplementary material for: Situating Meditation Apps Within the Ecosystem of Meditation Practice: Population-Based Survey Study
Source: JMIR Ment Health. 2023 Apr 28;10:e43565. doi: 10.2196/43565 (PMC10182467; doi:10.2196/43565)
Supplement: Multimedia Appendix 3 [file mental_v10i1e43565_app3.docx]

*Follow-up Survey Items*

1. Why did you **FIRST** try meditation? (select all that apply)
   1. Physical health
   2. Mental/emotional health or stress reduction
   3. Part of social, cultural, or religious identity
   4. General spiritual / self-transformation
   5. Enlightenment, awakening, nirvana, or other ultimate goal
   6. Other (please specify) (text field)
2. Please respond to each question or statement by clicking one box per row.

In the past 7 days…(1 = Never; 2 = Rarely; 3 = Sometimes; 4 = Usually; 5 = Always)

1. I felt worthless
2. I felt helpless
3. I felt depressed
4. I felt hopeless
5. Please respond to each question or statement by clicking one box per row. In the past 7 days…(1 = Never; 2 = Rarely; 3 = Sometimes; 4 = Usually; 5 = Always)
6. I felt fearful
7. I found it hard to focus on anything other than my anxiety
8. My worries overwhelmed me
9. I felt uneasy
10. For the next set of questions, please read each statement and then decide how much each applies to you in the past week. In the past week, please rate how often… (1 = Never; 2 = Rarely; 3 = Sometimes; 4 = Usually; 5 = Always)
11. I feel alone and apart from others
12. I feel left out
13. I feel that I am no longer close to anyone
14. I feel alone
15. I feel lonely
16. Which of the following meditation smartphone apps have you **EVER** used? (select all that apply)
    1. Headspace
    2. Calm
    3. Insight Timer
    4. Happify
    5. 10% Happier
    6. Healthy Minds Program
    7. Waking Up
    8. Smiling Mind
    9. Stop, Breathe, and Think
    10. Pacifica
    11. Simple Habit
    12. Breethe
    13. The Mindfulness App
    14. Aura
    15. 21-Day Meditation Experience
    16. Digipill: Guided Meditation
    17. Omvana
    18. Breathe2Relax
    19. Sattva
    20. Liberate
    21. Inscape
    22. Buddhify
    23. Waking Up
    24. Other (please specify) (text field)
    25. I have not used a meditation smartphone app
17. (If responded other than “x” to above question), Which of the following meditation smartphone apps have you used MOST? (select one)
18. (If responded other than “I have not used a meditation smartphone app”), How often do you use this app?
    1. Daily
       1. Please provide minutes per day
    2. Weekly
       1. Please provide minutes per week
    3. Monthly
       1. Please provide minutes per month
    4. Several times per year
       1. Please provide minutes per year
    5. Never
19. Which of the following concerns do you have about meditation apps? (select all that apply)
    1. Cost of apps
    2. Time required for use
    3. Unsure if they are effective
    4. Not recommended by a healthcare provider
    5. I am not interested in them
    6. My health data is not secure
    7. They do not target or help with my goals
    8. Not user friendly
    9. I experienced technical problems
    10. Apps are not a trustworthy source of information
    11. Other (please specify) (text response)
    12. I do not have any concerns about meditation apps

9. How much would you be interested in the following meditation smartphone app features?
(1 = not at all, 6 = a great deal)

- 1. Ability to connect with other users
  2. Ability to link app use to social media (e.g., Facebook, Twitter, Instagram, TikTok)
  3. Complete questionnaires that track my mood to customize practices
  4. Automated feedback based on sensors in my phone (e.g., location, text messages, camera)
  5. Content related to depression and anxiety
  6. Encourage me to try “mini” meditation practices based on my mood
  7. Ability to text with a meditation coach
  8. Ability to speak with a meditation coach by phone / videocall
  9. Ability to attend live “classes”
  10. Rewards (e.g., trophies) for practicing a certain number of days
  11. Opportunities to journal / complete reflections about my experience
  12. Tips for daily life practice
  13. Ability to set myself reminders to practice
