# Supplementary material for: Situating Meditation Apps Within the Ecosystem of Meditation Practice: Population-Based Survey Study
Source: JMIR Ment Health. 2023 Apr 28;10:e43565. doi: 10.2196/43565 (PMC10182467; doi:10.2196/43565)
Supplement: Multimedia Appendix 4 [file mental_v10i1e43565_app4.docx]

*Descriptive Statistics for Meditators (i.e., Lifetime Exposure to Meditation) Who Completed the Follow-Up Survey (n = 434)*

| Variables | mean | sd | % | N | min | max | skew | kurtosis |
| --- | --- | --- | --- | --- | --- | --- | --- | --- |
| Depression | 8.53 | 4.26 |  |  | 4 | 20 | 0.7 | -0.5 |
| Anxiety | 9.24 | 4.01 |  |  | 4 | 20 | 0.48 | -0.61 |
| Loneliness | 2.39 | 1.09 |  |  | 1 | 5 | 0.42 | -0.79 |
| Motivation for meditation |  |  |  |  |  |  |  |  |
| Physical health |  |  | 74.19 | 322 | 0 | 1 |  |  |
| Mental health |  |  | 15.90 | 69 | 0 | 1 |  |  |
| Cultural |  |  | 11.75 | 51 | 0 | 1 |  |  |
| Spiritual |  |  | 28.11 | 122 | 0 | 1 |  |  |
| Awakening |  |  | 14.74 | 64 | 0 | 1 |  |  |
| Concerns about meditation app |  |  |  |  |  |  |  |  |
| Total concerns | 1.95 | 1.12 |  |  | 1 | 7 | 1.21 | 1.26 |
| Cost |  |  | 42.40 | 184 | 0 | 1 |  |  |
| Time |  |  | 21.20 | 92 | 0 | 1 |  |  |
| Effectiveness |  |  | 35.48 | 154 | 0 | 1 |  |  |
| Recommendation | |  | 3.00 | 13 | 0 | 1 |  |  |
| Interest |  |  | 20.51 | 89 | 0 | 1 |  |  |
| Security |  |  | 12.67 | 55 | 0 | 1 |  |  |
| Target |  |  | 10.83 | 47 | 0 | 1 |  |  |
| Usability |  |  | 6.68 | 29 | 0 | 1 |  |  |
| Techprob |  |  | 4.38 | 19 | 0 | 1 |  |  |
| Trust |  |  | 8.29 | 36 | 0 | 1 |  |  |
| Desired meditation app features |  |  |  |  |  |  |  |  |
| Tips | 4.28 | 1.53 |  |  | 1 | 6 | -0.73 | -0.44 |
| Reminders | 4.19 | 1.67 |  |  | 1 | 6 | -0.62 | -0.86 |
| Mood-Based mini practice | 4.19 | 1.60 |  |  | 1 | 6 | -0.65 | -0.62 |
| Dep/Anx content | 3.94 | 1.71 |  |  | 1 | 6 | -0.48 | -1.06 |
| Track mood | 3.77 | 1.65 |  |  | 1 | 6 | -0.29 | -1.06 |
| Journaling | 3.40 | 1.70 |  |  | 1 | 6 | -0.01 | -1.25 |
| Rewards | 3.12 | 1.82 |  |  | 1 | 6 | 0.21 | -1.37 |
| Attend live class | 3.08 | 1.76 |  |  | 1 | 6 | 0.21 | -1.35 |
| Text coach | 3.00 | 1.75 |  |  | 1 | 6 | 0.29 | -1.27 |
| Sensor feedback | 2.67 | 1.67 |  |  | 1 | 6 | 0.48 | -1.16 |
| Speak with coach | 2.60 | 1.68 |  |  | 1 | 6 | 0.63 | -0.92 |
| Connect with others | 2.26 | 1.57 |  |  | 1 | 6 | 0.95 | -0.44 |
| Link social media | 1.72 | 1.27 |  |  | 1 | 6 | 1.75 | 2.10 |

*Note.* Skew and kurtosis values are not reported for dichotomous variables. Mental health motivation = practice motivation for mental/emotional health or stress reduction; Cultural motivation = practice motivation for social, cultural, or religious identity; Spiritual motivation = practice motivation for general spiritual / self-transformation; Awakening motivation = practice motivation for enlightenment, awakening, nirvana, or other ultimate goal; Total concerns = the total list of expressed concerns; Effectiveness Concern = unsure if apps are effective; Recommendation concern = concern of not recommended by a healthcare provider; Interest Concern = not interested in using apps; Security concern = concern of the security of health data; Target concern = concern of not targeting or helping with personal goals; Usability Concern = apps not user friendly; Techprob concerns = concerns of experiencing technical problems; Trust concern = concern of apps not being a trustworthy source of information. Tips = automated feedback based on sensors in my phone (e.g., location, text messages, camera); Reminders = ability to set practice reminders; Mood-Based Mini Practice = encourage me to try “mini” meditation practices based on my mood; Dep/Anx Content = content related to depression and anxiety; Track Mood = complete questionnaires that track my mood to customize practices; Journaling = opportunities to journal / complete reflections about my experience; Rewards = rewards (e.g., trophies) for practicing a certain number of days; Text Coach = ability to text with a meditation coach; Sensor Feedback = automated feedback based on sensors in my phone (e.g., location, text messages, camera); Speak with Coach = ability to speak with a meditation coach; Connect with Others = ability to connect with other users; Link with Social Media = ability to link app use to social media.
